# Interaction between the 5-HT system and the basal ganglia: functional implication and therapeutic perspective in Parkinson's disease

**DOI:** 10.3389/fncir.2014.00021

**Published:** 2014-03-17

**Authors:** Cristina Miguelez, Teresa Morera-Herreras, Maria Torrecilla, Jose A. Ruiz-Ortega, Luisa Ugedo

**Affiliations:** ^1^Department of Pharmacology, Faculty of Medicine and Dentistry, University of the Basque Country UPV/EHULeioa, Spain; ^2^Department of Pharmacology, Faculty of Pharmacy, University of the Basque Country UPV/EHUVitoria-Gasteiz, Spain

**Keywords:** 5-HT, basal ganglia, electrophysiology, Parkinson's disease, L-DOPA induced dyskinesia

## Abstract

The neurotransmitter serotonin (5-HT) has a multifaceted function in the modulation of information processing through the activation of multiple receptor families, including G-protein-coupled receptor subtypes (5-HT_1_, 5-HT_2_, 5-HT_4–7_) and ligand-gated ion channels (5-HT_3_). The largest population of serotonergic neurons is located in the midbrain, specifically in the raphe nuclei. Although the medial and dorsal raphe nucleus (DRN) share common projecting areas, in the basal ganglia (BG) nuclei serotonergic innervations come mainly from the DRN. The BG are a highly organized network of subcortical nuclei composed of the *striatum* (caudate and putamen), *subthalamic nucleus* (STN), internal and external *globus pallidus* (or entopeduncular nucleus in rodents, GPi/EP and GPe) and *substantia nigra* (*pars compacta*, SNc, and *pars reticulata*, SNr). The BG are part of the cortico-BG-thalamic circuits, which play a role in many functions like motor control, emotion, and cognition and are critically involved in diseases such as Parkinson's disease (PD). This review provides an overview of serotonergic modulation of the BG at the functional level and a discussion of how this interaction may be relevant to treating PD and the motor complications induced by chronic treatment with L-DOPA.

Serotonergic innervation in the brain originates from the raphe nuclei. Both, the medial and the dorsal raphe nucleus (DRN), project to common areas implicated in motor control, such as the thalamus. Nevertheless, the basal ganglia (BG) nuclei receive serotonergic afferences coming prevalently from the DRN (reviewed in Di Matteo et al., 2008). The BG contain serotonin (5-HT) and its metabolite 5-hydroxy-indolacetic acid (5-HIAA) (Palkovits et al., 1974; Saavedra, 1977; Lavoie and Parent, 1990), 5-HT transporter (SERT) and serotonergic receptors (from 5-HT_1_ to 5-HT_7_). These serotonergic receptors are unevenly expressed along the BG, and their distribution also differs between species. Here, we will review the evidences supporting the serotonergic system as a modulator of the BG functionality. Both physiological and pathological conditions will be analyzed from the basic and clinical point of view.

## Physiological serotonergic modulation of the basal ganglia

In accordance with its neuroanatomical distribution (as summarized in Table 1), 5-HT physiologically modulates BG nuclei activity by acting on serotonergic receptors.

**Table 1:** **Localization and expression density of serotonergic receptors in the basal ganglia of healthy brains of rodents, monkeys and humans**.

	**GPe/GPi(EP)**	**Striatum**	**STN**	**SNc/SNr**	**References**
5-HT_1A_	+^r^	+^r^	+^r^	+^r^	^r^Lanfumey and Hamon, 2000
	+^m^	+^m^(matrix)	+^m^	+^m^	^m^Frechilla et al., 2001; ^m^Huot et al., 2012a
	+^h^	++^m^(striosome)			
		+^h^			^h^Huot et al., 2012b
5-HT_1B_	+++^r^	++^r^	+++^r^	+++^r^	^r^Bruinvels et al., 1993
	+++^h^	++^h^		+++^h^	^h^Varnas et al., 2004a
5-HT_2A_	++^r^	++^r^	+^h^	+^r^	^r^Pazos et al., 1985
	+^m^	+^m^		+^m^	^m^Huot et al., 2012c
	++/+^h^	++^h^		++/+^h^	^h^Hoyer et al., 1986; ^h^Pazos et al., 1987; ^h^Hall et al., 2000; ^h^Varnas et al., 2004a
5-HT_2C_	++^r^	++^r^	+++^r^	+++^r^(c)	^r^Mengod et al., 1990; ^r^Pompeiano et al., 1994; ^r^Abramowski et al., 1995; ^r^Clemett et al., 2000
	+++^h^	+++^h^		+++^h^	^h^Pazos et al., 1987; ^h^Lopez-Gimenez et al., 2001
5-HT_3_		+^r^		+^r^	^r^Kilpatrick et al., 1987; ^r^Gehlert et al., 1993
		+++^h^		++^h^	^h^Bufton et al., 1993
5-HT_4_	+++^r^	+++^r^		+++^r^	^r,m^Jakeman et al., 1994; ^r^Nirogi et al., 2013
	+++^m^	+++^m^		+++^m^	
	+++^h^	+++^h^		+++^h^	^h^Bonaventure et al., 2000; ^h^Varnas et al., 2003, 2004a
5-HT_5A_	+^r^	+^r^	++^r^	+++^r^	^r^Oliver et al., 2000
5-HT_6_		+++^r^		++^r^	^r^Gerard et al., 1997
		+++^h^		++^h^	^h^Kohen et al., 1996
5-HT_7_	+^r^	+^r^	+^h^	+^r^	^r^Horisawa et al., 2013
		+^h^		+^h^	^r,h^Martin-Cora and Pazos, 2004
					^h^Varnas et al., 2004b

*+++, strong; ++, moderate; +, weak/r, rodent; m, monkey; h, human. EP, entopeduncular nucleus; GPe, external segment of the globus pallidus; GPi, internal segment of the globus pallidus; STN, subthalamic nucleus; SNc, substantia nigra pars compacta; SNr, substantia nigra pars reticulata*.

### Striatum

The striatum is the main input nucleus of the BG and a key neural substrate for motor function. Several studies have shown that 5-HT affects striatal function. In fact, both DRN stimulation and local administration of 5-HT into the striatum inhibit the vast majority of the striatal cells (Olpe and Koella, 1977; Davies and Tongroach, 1978; Yakel et al., 1988). However, by performing intracellular recordings, some researchers have reported striatal excitatory postsynaptic potentials after DRN stimulation, as well as a 5-HT-induced increase in firing rate of medium spiny neurons (MSN) (Vandermaelen et al., 1979; Park et al., 1982; Stefani et al., 1990; Wilms et al., 2001). Stimulation of presynaptic 5-HT_1A_ and 5-HT_1B_ receptors inhibits striatal 5-HT release (Gerber et al., 1988; Knobelman et al., 2000), and these receptors also control the release of other neurotransmitters in the striatum. Accordingly, 5-HT_1A_ receptor activation decreases glutamate release from corticostriatal projections (Antonelli et al., 2005; Mignon and Wolf, 2005; Dupre et al., 2011, 2013). On the other hand, activation of 5-HT_1B_ receptors indirectly stimulates the *substantia nigra pars compacta* (SNc) by decreasing GABA release from the *substantia nigra pars reticulata* (SNr), what consequently leads to increasing striatal dopamine levels (Gerber et al., 1988).

The 5-HT_2_ receptor family produces an inhibitory action on striatal neuron activity, mainly by modulating MSN (el Mansari et al., 1994; el Mansari and Blier, 1997). Moreover, Rueter et al. (2000) have shown that 5-HT_2C_ receptors exert tonic inhibitory control over MSN membrane excitability. Other *in vivo* studies, however, have shown contradictory results suggesting that the effect of serotonergic drugs depends on the area of the striatum analyzed (Wilms et al., 2001). 5-HT_2_ receptor activation indirectly reduces the activity of striatal MSN by enhancing the inhibitory tone of cholinergic interneurons over these output neurons. The increased release of acetylcholine is due to activation of cholinergic interneurons mainly through 5-HT_2C_ receptors, although the involvement of 5-HT_6_ and 5-HT_7_ receptors has also been demonstrated (Bonsi et al., 2007; Blomeley and Bracci, 2009). In addition, the activation of 5-HT_2C_ receptors located on fast-spiking interneurons increases their excitability, causing an enhancement of GABAergic postsynaptic inhibition that also decreases the activity of striatal projecting neurons (Blomeley and Bracci, 2009).

### Subthalamic nucleus

5-HT exerts a complex effect in the *subthalamic nucleus* (STN) that is considered to be a powerful excitatory drive in the BG motor circuit. Both pharmacological lesion of the DRN and 5-HT depletion increase STN firing frequency and burst activity *in vivo* (Liu et al., 2007; Aristieta et al., 2013). Decreased and increased excitability have been reported with the activation of 5-HT_1A_ and 5-HT_2C_, and 5-HT_4_ receptors, respectively (Flores et al., 1995; Stanford et al., 2005; Xiang et al., 2005; Shen et al., 2007; Aristieta et al., 2013). In addition, activation of 5-HT_1B_ receptors inhibits synaptic activity of STN neurons (Barwick et al., 2000; Shen and Johnson, 2008).

### Globus pallidus

The *globus pallidus* (GP) has two segments, the external GP (GPe), which has a central position in the BG loop, and the internal GP (GPi/EP), which, together with the SNr, form the output structures of the BG. In the GPe, 5-HT depletion decreases the firing frequency and increases the proportion of bursty and irregular neurons (Delaville et al., 2012b). In contrast, local application of 5-HT or selective serotonin reuptake inhibitor (SSRI) administration excites most of GPe neurons (Querejeta et al., 2005; Zhang et al., 2010; Wang et al., 2013). These findings have been further confirmed by a patch-clamp recording study in which 5-HT perfusion produced a reversible depolarization of the GP neuron membrane potential, thereby increasing the firing rate of these neurons (Chen et al., 2008). *In vivo* studies indicate that the stimulatory effect of 5-HT on GPe neurons is mediated by the activation of 5-HT_4_ or 5-HT_7_ postsynaptic receptors, but not 5-HT_2C_ and 5-HT_3_ receptors (Bengtson et al., 2004; Kita et al., 2007; Chen et al., 2008; Hashimoto and Kita, 2008). In contrast, 5-HT can decrease the presynaptic release of glutamate and GABA from the subthalamopallidal and striatopallidal terminals, respectively, through 5-HT_1B_ receptors (Querejeta et al., 2005). In addition, 5-HT has been proposed to modulate the inhibitory and excitatory responses in GPe electrical stimulation of the motor cortex in awake monkeys (Kita et al., 2007). In fact, 5-HT suppresses GABAergic inhibitory responses to cortical stimulation through presynaptic 5-HT_1B_ receptors and glutamatergic excitatory responses involving presynaptic or postsynaptic 5-HT_1A_ receptors (Kita et al., 2007).

Few studies have been conducted to investigate the effects of 5-HT on the GPi/EP nucleus. Recently, it has been shown that intra-EP administration of a 5-HT_2_ receptor agonist promotes oral movements and inhibits EP neuronal activity in dopamine-depleted rats (Lagiere et al., 2013).

### Substantia nigra

Together with the GPi, the SNr constitutes the principal output nucleus of the BG and plays a relevant role in movement initiation. In this nucleus, 5-HT induces mostly an inhibitory effect *in vivo* (Dray et al., 1976; Collingridge and Davies, 1981), while 5-HT depletion decreases firing rate and increases burst activity of SNr neurons (Delaville et al., 2012a). Electrophysiological studies carried out in brain slices indicate that 5-HT not only excites SNr neurons acting directly on 5-HT_2C_ receptors (Rick et al., 1995; Stanford and Lacey, 1996; Stanford et al., 2005) but also disinhibits SNr neurons by reducing GABA release from striatonigral terminals via presynaptic 5-HT_1B_ receptor stimulation (Stanford and Lacey, 1996). A recent electrophysiological study reveals that presynaptic 5-HT_1B_ receptor activation gates STN excitatory inputs to the SNr and reduces burst firing activity of the SNr, and therefore may be critically involved in movement control (Ding et al., 2013).

The role of 5-HT transmission in modulating the activity of dopaminergic SNc neurons is still unclear. Although the effect of 5-HT input seems to be inhibitory (Sinton and Fallon, 1988; Arborelius et al., 1993), chemical lesion of the DRN does not significantly alter SNc activity and DRN electrical stimulation only inhibits spontaneous activity in a subset of neurons (Kelland et al., 1990). Further, SSRI administration does not modulate SNc activity (Prisco and Esposito, 1995), and 5-HT depletion has been shown to either decrease or have no significant effect on SNc neuron excitability (Kelland et al., 1990; Minabe et al., 1996). Non-selective 5-HT_2_ receptor antagonists stimulate SNc neurons (Ugedo et al., 1989), whereas 5-HT_4_ receptors selectively prevents the stimulatory effect induced by haloperidol in this brain area (Lucas et al., 2001).

## Implication of the serotonergic system in Parkinson's disease

In the parkinsonian state and subsequent replacement therapy with L-DOPA, the serotonergic system adapts to the lack of dopamine by adopting anatomical and functional transformations.

### Serotonergic system in Parkinson's disease and parkinsonian animal models

**Figure 1:**
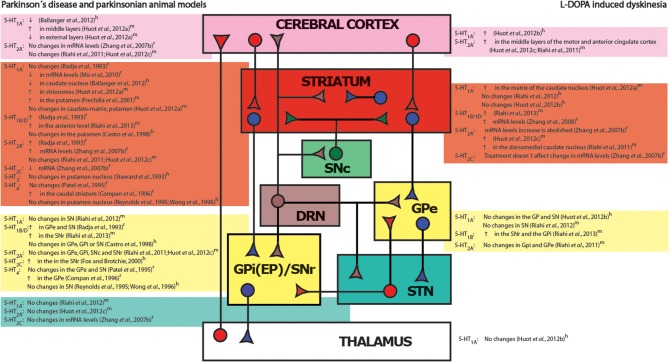
**Simplified diagram of the basal ganglia circuits and altered serotonergic receptor expression in pathological states**. Changes found in serotonergic receptor density in parkinsonian (left boxes) and dyskinetic (right boxes) patients or animals models compared to control conditions. Each nucleus and its modifications in receptor expression are encoded with the same color. GABAergic inhibitory pathways are represented in dark blue and glutamatergic excitatory pathways in red. Modulatory dopaminergic connections are indicated in green and serotonergic pathways in brown. DRN, *dorsal raphe nucleus*; GPi (EP), internal segment of the *globus pallidus (entopeduncular nucleus)*; GPe, external segment of the *globus pallidus*; STN, *subthalamic nucleus*; SNc, *substantia nigra pars compacta*; SNr, *substantia nigra pars reticulata*. r, rodent; m, monkey; h, human.

Parkinson's disease (PD) is a neurodegenerative disease typified by loss of dopaminergic neurons in the SNc and subsequent dopamine depletion in the striatum. In patients with PD, it is generally supported that serotonergic neurotransmission decreases in advanced stages of the disease (Haapaniemi et al., 2001; Kerenyi et al., 2003) since the DRN, in addition to other nuclei, undergoes degeneration (Halliday et al., 1990; Jellinger, 1990). Moreover, 5-HT and 5-HIAA concentrations, as well as SERT expression, are reduced in several BG nuclei (Scatton et al., 1983; Raisman et al., 1986; D'Amato et al., 1987; Chinaglia et al., 1993; Kerenyi et al., 2003; Guttman et al., 2007; Kish et al., 2008; Rylander et al., 2010). Regarding receptor expression, 5-HT_1A_ is decreased and 5-HT_2C_ is increased in some BG nuclei (Fox and Brotchie, 2000; Ballanger et al., 2012) (Figure 1). Other serotonergic receptor (5-HT_1B/D_, 5-HT_3_, and 5-HT_4_) densities are however not modified by the dopaminergic loss (Steward et al., 1993; Reynolds et al., 1995; Wong et al., 1996; Castro et al., 1998). Overall, this dysfunctional serotonergic neurotransmission can indeed be linked to the high prevalence of depressive symptoms in parkinsonian patients (Reijnders et al., 2008).

In animal models of parkinsonism, the changes occurring after dopaminergic lesion have not been equally reproduced by different research groups. The discrepancies between these studies may be due to different protocol paradigms used for inducing the parkinsonian state, including the age of the animals, site of injection, concentration of the toxin, and the time between surgery and performing the studies. Several researchers have reported hyperinnervation (Zhou et al., 1991; Rozas et al., 1998; Balcioglu et al., 2003; Maeda et al., 2003), while others found no sprouting (Prinz et al., 2013), or even a decrease in striatal serotonergic fibers after dopaminergic damage (Takeuchi et al., 1991; Rylander et al., 2010). Along the same lines, striatal 5-HT levels have been found to be increased (Commins et al., 1989; Zhou et al., 1991; Karstaedt et al., 1994; Balcioglu et al., 2003), unchanged (Breese et al., 1984; Carta et al., 2006), or decreased (Frechilla et al., 2001; Aguiar et al., 2006, 2008). As detailed in Figure 1, studies performed in different animal models report unequal modification in serotonergic receptor expression along the BG nuclei. On the other hand, the DRN also suffers adaptative changes after the dopaminergic degeneration, such as increased 5-HT_1A_ expression in MPTP monkeys (Frechilla et al., 2001) or weaker inhibitory effects of 5-HT_1A_ agonists on neuron activity in rats (Wang et al., 2009). Electrophysiological studies using different 6-hydroxydopamine (6-OHDA) lesion models have shown increased basal firing rate of serotonergic cells in the parkinsonian state (Zhang et al., 2007a; Kaya et al., 2008; Wang et al., 2009; Prinz et al., 2013), while others show decreases (Guiard et al., 2008) or no changes (Miguelez et al., 2011).

In spite of the disparity of results, it seems clear that to varying extents, the serotonergic system is affected in parkinsonian conditions. More clinical and preclinical studies using the same experimental models and a greater amount of samples would help to clarify the role of the serotonergic system in each stage of PD.

### Serotonergic system in L-DOPA induced dyskinesia

The dopamine precursor L-DOPA is the most effective pharmacological treatment for PD, but it does not stop the progression of the disease. Moreover, long-term administration of L-DOPA induces motor complications, known as L-DOPA induced dyskinesias (LID), which have been related to adaptive changes of the serotonergic system. For example, a recent publication revealed that patients who had developed dyskinetic movements showed significant serotonergic hyperinnervation in the GPe and caudate, in comparison to non-dyskinetic individuals (Rylander et al., 2010). Such sprouting was directly correlated with the severity of motor complications. In contrast, other studies have shown that striatal *postmortem* content of 5-HT and SERT levels did not differ significantly between dyskinetic and non-dyskinetic cases (Calon et al., 2003; Kish et al., 2008), and chronic L-DOPA treatment did not influence SERT expression (Politis et al., 2010). As for serotonergic receptors, a study performed in PD patients that followed L-DOPA treatment showed increased 5-HT_1A_ expression in several cortical areas, while no modification in the striatum, GP, SN, or thalamus was reported (Huot et al., 2012b). In the SNr, 5-HT_2C_ expression has also been observed to be raised in those patients (Fox and Brotchie, 2000).

The use of animal models has provided valuable data to better understand the physiopathological mechanisms of LID. The most used models include non-human primates injected with MPTP and rodent-models with hemilateral dopaminergic loss chronically treated with L-DOPA. Although differences may arise from the methodological protocols, such models are considered to reproduce resembling symptoms and molecular changes to those observed in PD patients and efficiently respond to antidyskinetic therapy (Iderberg et al., 2012). It is now well known that exogenously administered L-DOPA can be stored, transformed into dopamine, and released from serotonergic terminals to multiple brain regions, including the striatum, in an uncontrolled manner, producing a non-physiological stimulation of sensitized dopaminergic receptors (Arai et al., 1995; Carta et al., 2007; Yamada et al., 2007; Navailles et al., 2010b, 2013). Lesions of the DRN consistently prevent the expression of dyskinesia (Carta et al., 2007; Eskow et al., 2009) or dopamine release after an acute L-DOPA injection (Navailles et al., 2010b). This interaction between serotonergic and dopaminergic systems is reciprocal, as 5-HT levels also decrease after L-DOPA administration, and L-DOPA itself can antagonize the effect of serotonergic agents (Bartholini et al., 1968; Everett and Borcherding, 1970; Commissiong and Sedgwick, 1979; Borah and Mohanakumar, 2007; Navailles et al., 2010a; Riahi et al., 2011; Miguelez et al., 2013). In dyskinetic animals, SERT expression has been found to be up-regulated (Rylander et al., 2010), not modified (Prinz et al., 2013), or decreased (Nevalainen et al., 2011). Serotonergic receptor expression in the BG is unevenly modified with L-DOPA treatment: 5-HT_2A_ and 5-HT_1B_ receptor expression is increased (Zhang et al., 2008; Riahi et al., 2011, 2013; Huot et al., 2012c), while 5-HT_1A_ receptor expression is increased (Huot et al., 2012a) or does not change (Riahi et al., 2012) (Figure 1). The primary modifications occurring in the serotonergic system are thought to take place at terminal levels because no changes in the number of serotonergic neurons (Rylander et al., 2010; Inden et al., 2012) or 5-HT or dopamine levels in the DRN of dyskinetic rats have been reported (Bishop et al., 2012).

## Clinical relevance

Although motor complications appear in the majority of the patients that receive chronic treatment with L-DOPA, an effective pharmacological tool for avoiding or treating LID expression is still missing. In this sense, 5-HT_1A/1C_ receptors, which are involved in the regulation of the ectopic dopamine release, are envisaged as promising targets. In 6-OHDA-lesioned rats and MPTP monkeys chronically treated with L-DOPA, 5-HT_1A/1C_ receptor agonists reduce expression of LID without impairing L-DOPA improvement in motor performance (Bibbiani et al., 2001; Ba et al., 2007; Dupre et al., 2007). Furthermore, administration of the 5-HT_1A_ agonist, 8-OH-DPAT, also prevents L-DOPA-induced increment of extracellular dopamine (Nahimi et al., 2012). Other drugs that modulate 5-HT neurotransmission have shown efficacy over LID. Thus, a recent study has revealed that the treatment with the precursor of 5-HT, 5-hydroxytryptophan reduces the appearance of LID in L-DOPA-primed rats (Tronci et al., 2013). The 5-HT_2A_ receptor inverse agonist ACP-103 reduces tremor in rodents and LID in MPTP monkeys (Vanover et al., 2008). Acute and prolonged SSRI treatment attenuates the severity and development of LID in L-DOPA-primed and naive rats without interfering with motor improvement, which may be mediated in part by 5-HT_1A_ receptors (Bishop et al., 2012; Conti et al., 2014). In contrast, in PD patients, while buspirone, a partial 5-HT_1A_ agonist, ameliorates dyskinesia (Kleedorfer et al., 1991; Bonifati et al., 1994), sarizotan, another 5-HT_1A_ receptor agonist, failed to improve it compared with placebo (Goetz et al., 2008) and significantly increased *off* time (Goetz et al., 2007).

## Concluding remarks

The effects of 5-HT in the BG depend on the specific nucleus and its receptor distribution. 5-HT induces an inhibition of MSN in the striatum using either direct or indirect activation of serotonergic receptors, as well as in the STN and SNr *in vivo*. In contrast, in the GPe the overall effect of 5-HT is excitatory. In other nuclei such as the EP or SNc the net effect is still not well understood.

The serotonergic physiological modulation may be modified in pathological conditions where the BG nuclei are highly affected. Here, we provide data regarding the alteration of the serotonergic system in PD, pointing out important discrepancies about the relationship between the serotonergic and dopaminergic systems in pathological states. In this concern, key methodological differences such as the use of different animal species and models, pharmacological treatments or stage of the disease in PD patients may explain these inconsistencies.

In summary, the serotonergic system is implicated in the modulation of the BG activity and in the etiopathology of PD and LID. However, although in preclinical studies results indicate that serotonergic drugs may be suitable for treating LID, this fact has yet to be supported by clinical trials. Accordingly, further investigation is required to determine the most suitable serotonergic target to treat these motor disturbances.

### Conflict of interest statement

The authors declare that the research was conducted in the absence of any commercial or financial relationships that could be construed as a potential conflict of interest.

## References

[B1] Abramowski D. Rigo M. Duc D. Hoyer D. Staufenbiel M. (1995). Localization of the 5-hydroxytryptamine2C receptor protein in human and rat brain using specific antisera. Neuropharmacology 34, 1635–1645 10.1016/0028-3908(95)00138-7 8788961

[B2] Aguiar L. M. Macedo D. S. Vasconcelos S. M. Oliveira A. A. De Sousa F. C. Viana G. S. (2008). CSC, an adenosine A(2A) receptor antagonist and MAO B inhibitor, reverses behavior, monoamine neurotransmission, and amino acid alterations in the 6-OHDA-lesioned rats. Brain Res. 1191, 192–199 10.1016/j.brainres.2007.11.051 18164694

[B3] Aguiar L. M. Nobre H. V. Jr. Macedo D. S. Oliveira A. A. Freitas R. M. Vasconcelos S. M. (2006). Neuroprotective effects of caffeine in the model of 6-hydroxydopamine lesion in rats. Pharmacol. Biochem. Behav. 84, 415–419 10.1016/j.pbb.2006.05.027 16844208

[B4] Antonelli T. Fuxe K. Tomasini M. C. Bartoszyk G. D. Seyfried C. A. Tanganelli S. (2005). Effects of sarizotan on the corticostriatal glutamate pathways. Synapse 58, 193–199 10.1002/syn.20195 16138317

[B5] Arai R. Karasawa N. Geffard M. Nagatsu I. (1995). L-DOPA is converted to dopamine in serotonergic fibers of the striatum of the rat: a double-labeling immunofluorescence study. Neurosci. Lett. 195, 195–198 10.1016/0304-3940(95)11817-G 8584208

[B6] Arborelius L. Chergui K. Murase S. Nomikos G. G. Hook B. B. Chouvet G. (1993). The 5-HT1A receptor selective ligands, (R)-8-OH-DPAT and (S)-UH-301, differentially affect the activity of midbrain dopamine neurons. Naunyn Schmiedebergs Arch. Pharmacol. 347, 353–362 10.1007/BF00165384 8510763

[B7] Aristieta A. Morera-Herreras T. Ruiz-Ortega J. A. Miguelez C. Vidaurrazaga I. Arrue A. (2013). Modulation of the subthalamic nucleus activity by serotonergic agents and fluoxetine administration. Psychopharmacology (Berl.). [Epub ahead of print]. 10.1007/s00213-013-3333-0 24271033 PMC3984421

[B8] Ba M. Kong M. Ma G. Yang H. Lu G. Chen S. (2007). Cellular and behavioral effects of 5-HT1A receptor agonist 8-OH-DPAT in a rat model of levodopa-induced motor complications. Brain Res. 1127, 177–184 10.1016/j.brainres.2006.10.020 17113046

[B9] Balcioglu A. Zhang K. Tarazi F. I. (2003). Dopamine depletion abolishes apomorphine- and amphetamine-induced increases in extracellular serotonin levels in the striatum of conscious rats: a microdialysis study. Neuroscience 119, 1045–1053 10.1016/S0306-4522(03)00219-7 12831863

[B10] Ballanger B. Klinger H. Eche J. Lerond J. Vallet A. E. Le Bars D. (2012). Role of serotonergic 1A receptor dysfunction in depression associated with Parkinson's disease. Mov. Disord. 27, 84–89 10.1002/mds.23895 21994070

[B11] Bartholini G. Da Prada M. Pletscher A. (1968). Decrease of cerebral 5-hydroxytryptamine by 3,4-dihydroxyphenylalanine after inhibition of extracerebral decarboxylase. J. Pharm. Pharmacol. 20, 228–229 10.1111/j.2042-7158.1968.tb09726.x 4385052

[B12] Barwick V. S. Jones D. H. Richter J. T. Hicks P. B. Young K. A. (2000). Subthalamic nucleus microinjections of 5-HT2 receptor antagonists suppress stereotypy in rats. Neuroreport 11, 267–270 10.1097/00001756-200002070-00009 10674468

[B13] Bengtson C. P. Lee D. J. Osborne P. B. (2004). Opposing electrophysiological actions of 5-HT on noncholinergic and cholinergic neurons in the rat ventral pallidum *in vitro*. J. Neurophysiol. 92, 433–443 10.1152/jn.00543.2003 14960557

[B14] Bibbiani F. Oh J. D. Chase T. N. (2001). Serotonin 5-HT1A agonist improves motor complications in rodent and primate parkinsonian models. Neurology 57, 1829–1834 10.1212/WNL.57.10.1829 11723272

[B15] Bishop C. George J. A. Buchta W. Goldenberg A. A. Mohamed M. Dickinson S. O. (2012). Serotonin transporter inhibition attenuates l-DOPA-induced dyskinesia without compromising l-DOPA efficacy in hemi-parkinsonian rats. Eur. J. Neurosci. 36, 2839–2848 10.1111/j.1460-9568.2012.08202.x 22762478 PMC3445783

[B16] Blomeley C. P. Bracci E. (2009). Serotonin excites fast-spiking interneurons in the striatum. Eur. J. Neurosci. 29, 1604–1614 10.1111/j.1460-9568.2009.06725.x 19419423 PMC2695856

[B17] Bonaventure P. Hall H. Gommeren W. Cras P. Langlois X. Jurzak M. (2000). Mapping of serotonin 5-HT(4) receptor mRNA and ligand binding sites in the post-mortem human brain. Synapse 36, 35–46 10.1002/(SICI)1098-2396(200004)36:1<35::AID-SYN4>3.0.CO;2-Y 10700024

[B18] Bonifati V. Fabrizio E. Cipriani R. Vanacore N. Meco G. (1994). Buspirone in levodopa-induced dyskinesias. Clin. Neuropharmacol. 17, 73–82 10.1097/00002826-199402000-00008 8149361

[B19] Bonsi P. Cuomo D. Ding J. Sciamanna G. Ulrich S. Tscherter A. (2007). Endogenous serotonin excites striatal cholinergic interneurons via the activation of 5-HT 2C, 5-HT6, and 5-HT7 serotonin receptors: implications for extrapyramidal side effects of serotonin reuptake inhibitors. Neuropsychopharmacology 32, 1840–1854 10.1038/sj.npp.1301294 17203014

[B20] Borah A. Mohanakumar K. P. (2007). Long-term L-DOPA treatment causes indiscriminate increase in dopamine levels at the cost of serotonin synthesis in discrete brain regions of rats. Cell. Mol. Neurobiol. 27, 985–996 10.1007/s10571-007-9213-6 17934805 PMC11517132

[B21] Breese G. R. Baumeister A. A. McCown T. J. Emerick S. G. Frye G. D. Crotty K. (1984). Behavioral differences between neonatal and adult 6-hydroxydopamine-treated rats to dopamine agonists: relevance to neurological symptoms in clinical syndromes with reduced brain dopamine. J. Pharmacol. Exp. Ther. 231, 343–354 6149306 PMC3060042

[B22] Bruinvels A. T. Palacios J. M. Hoyer D. (1993). Autoradiographic characterisation and localisation of 5-HT1D compared to 5-HT1B binding sites in rat brain. Naunyn Schmiedebergs Arch. Pharmacol. 347, 569–582 10.1007/BF00166939 8361548

[B23] Bufton K. E. Steward L. J. Barber P. C. Barnes N. M. (1993). Distribution and characterization of the [3H]granisetron-labelled 5-HT3 receptor in the human forebrain. Neuropharmacology 32, 1325–1331 10.1016/0028-3908(93)90027-Z 8152523

[B24] Calon F. Morissette M. Rajput A. H. Hornykiewicz O. Bedard P. J. Di Paolo T. (2003). Changes of GABA receptors and dopamine turnover in the postmortem brains of parkinsonians with levodopa-induced motor complications. Mov. Disord. 18, 241–253 10.1002/mds.10343 12621627

[B25] Carta M. Carlsson T. Kirik D. Bjorklund A. (2007). Dopamine released from 5-HT terminals is the cause of L-DOPA-induced dyskinesia in parkinsonian rats. Brain 130, 1819–1833 10.1093/brain/awm082 17452372

[B26] Carta M. Lindgren H. S. Lundblad M. Stancampiano R. Fadda F. Cenci M. A. (2006). Role of striatal L-DOPA in the production of dyskinesia in 6-hydroxydopamine lesioned rats. J. Neurochem. 96, 1718–1727 10.1111/j.1471-4159.2006.03696.x 16539687

[B27] Castro M. E. Pascual J. Romon T. Berciano J. Figols J. Pazos A. (1998). 5-HT1B receptor binding in degenerative movement disorders. Brain Res. 790, 323–328 10.1016/S0006-8993(97)01566-7 9593971

[B28] Chen L. Yung K. K. Chan Y. S. Yung W. H. (2008). 5-HT excites globus pallidus neurons by multiple receptor mechanisms. Neuroscience 151, 439–451 10.1016/j.neuroscience.2007.11.003 18082329

[B29] Chinaglia G. Landwehrmeyer B. Probst A. Palacios J. M. (1993). Serotoninergic terminal transporters are differentially affected in Parkinson's disease and progressive supranuclear palsy: an autoradiographic study with [3H]citalopram. Neuroscience 54, 691–699 10.1016/0306-4522(93)90240-G 8332256

[B30] Clemett D. A. Punhani T. Duxon M. S. Blackburn T. P. Fone K. C. (2000). Immunohistochemical localisation of the 5-HT2C receptor protein in the rat CNS. Neuropharmacology 39, 123–132 10.1016/S0028-3908(99)00086-6 10665825

[B31] Collingridge G. L. Davies J. (1981). The influence of striatal stimulation and putative neurotransmitters on identified neurones in the rat substantia nigra. Brain Res. 212, 345–359 10.1016/0006-8993(81)90467-4 6112050

[B32] Commins D. L. Shaughnessy R. A. Axt K. J. Vosmer G. Seiden L. S. (1989). Variability among brain regions in the specificity of 6-hydroxydopamine (6-OHDA)-induced lesions. J. Neural Transm. 77, 197–210 10.1007/BF01248932 2503586

[B33] Commissiong J. W. Sedgwick E. M. (1979). Depletion of 5-HT by L-DOPA in spinal cord and brainstem of rat. Life Sci. 25, 83–86 10.1016/0024-3205(79)90493-4 481125

[B34] Compan V. Daszuta A. Salin P. Sebben M. Bockaert J. Dumuis A. (1996). Lesion study of the distribution of serotonin 5-HT4 receptors in rat basal ganglia and hippocampus. Eur. J. Neurosci. 8, 2591–2598 10.1111/j.1460-9568.1996.tb01553.x 8996808

[B35] Conti M. M. Ostock C. Y. Lindenbach D. Goldenberg A. A. Kampton E. Dell'isola R. (2014). Effects of prolonged selective serotonin reuptake inhibition on the development and expression of l-DOPA-induced dyskinesia in hemi-parkinsonian rats. Neuropharmacology 77, 1–8 10.1016/j.neuropharm.2013.09.017 24067924 PMC3865178

[B36] D'Amato R. J. Zweig R. M. Whitehouse P. J. Wenk G. L. Singer H. S. Mayeux R. (1987). Aminergic systems in Alzheimer's disease and Parkinson's disease. Ann. Neurol. 22, 229–236 10.1002/ana.410220207 3477996

[B37] Davies J. Tongroach P. (1978). Neuropharmacological studies on the nigro-striatal and raphe-striatal system in the rat. Eur. J. Pharmacol. 51, 91–100 10.1016/0014-2999(78)90333-3 29766

[B38] Delaville C. Chetrit J. Abdallah K. Morin S. Cardoit L. De Deurwaerdere P. (2012a). Emerging dysfunctions consequent to combined monoaminergic depletions in Parkinsonism. Neurobiol. Dis. 45, 763–773 10.1016/j.nbd.2011.10.023 22079236

[B39] Delaville C. Navailles S. Benazzouz A. (2012b). Effects of noradrenaline and serotonin depletions on the neuronal activity of globus pallidus and substantia nigra pars reticulata in experimental parkinsonism. Neuroscience 202, 424–433 10.1016/j.neuroscience.2011.11.024 22138505

[B40] Di Matteo V. Pierucci M. Esposito E. Crescimanno G. Benigno A. Di Giovanni G. (2008). Serotonin modulation of the basal ganglia circuitry: therapeutic implication for Parkinson's disease and other motor disorders. Prog. Brain Res. 172, 423–463 10.1016/S0079-6123(08)00921-7 18772045

[B41] Ding S. Li L. Zhou F. M. (2013). Presynaptic serotonergic gating of the subthalamonigral glutamatergic projection. J. Neurosci. 33, 4875–4885 10.1523/JNEUROSCI.4111-12.2013 23486958 PMC3617555

[B42] Dray A. Gonye T. J. Oakley N. R. (1976). Caudate stimulation and substantia nigra activity in the rat. J. Physiol. 259, 825–849 8637 10.1113/jphysiol.1976.sp011497 PMC1309066

[B43] Dupre K. B. Eskow K. L. Negron G. Bishop C. (2007). The differential effects of 5-HT(1A) receptor stimulation on dopamine receptor-mediated abnormal involuntary movements and rotations in the primed hemiparkinsonian rat. Brain Res. 1158, 135–143 10.1016/j.brainres.2007.05.005 17553470

[B44] Dupre K. B. Ostock C. Y. Eskow Jaunarajs K. L. Button T. Savage L. M. (2011). Local modulation of striatal glutamate efflux by serotonin 1A receptor stimulation in dyskinetic, hemiparkinsonian rats. Exp. Neurol. 229, 288–299 10.1016/j.expneurol.2011.02.012 21352823 PMC3100430

[B45] Dupre K. B. Ostock C. Y. George J. A. Eskow Jaunarajs K. L. Hueston C. M. Bishop C. (2013). Effects of 5-HT1A receptor stimulation on D1 receptor agonist-induced striatonigral activity and dyskinesia in hemiparkinsonian rats. ACS Chem. Neurosci. 4, 747–760 10.1021/cn300234z 23496922 PMC3656750

[B46] el Mansari M. Blier P. (1997). *In vivo* electrophysiological characterization of 5-HT receptors in the guinea pig head of caudate nucleus and orbitofrontal cortex. Neuropharmacology 36, 577–588 10.1016/S0028-3908(97)00035-X 9225283

[B47] el Mansari M. Radja F. Ferron A. Reader T. A. Molina-Holgado E. Descarries L. (1994). Hypersensitivity to serotonin and its agonists in serotonin-hyperinnervated neostriatum after neonatal dopamine denervation. Eur. J. Pharmacol. 261, 171–178 10.1016/0014-2999(94)90316-6 8001641

[B48] Eskow K. L. Dupre K. B. Barnum C. J. Dickinson S. O. Park J. Y. Bishop C. (2009). The role of the dorsal raphe nucleus in the development, expression, and treatment of L-dopa-induced dyskinesia in hemiparkinsonian rats. Synapse 63, 610–620 10.1002/syn.20630 19309758 PMC2741636

[B49] Everett G. M. Borcherding J. W. (1970). L-DOPA: effect on concentrations of dopamine, norepinephrine, and serotonin in brains of mice. Science 168, 847–850 10.1126/science.168.3933.849 5444060

[B50] Flores G. Rosales M. G. Hernandez S. Sierra A. Aceves J. (1995). 5-Hydroxytryptamine increases spontaneous activity of subthalamic neurons in the rat. Neurosci. Lett. 192, 17–20 10.1016/0304-3940(95)11597-P 7675300

[B51] Fox S. H. Brotchie J. M. (2000). 5-HT2C receptor binding is increased in the substantia nigra pars reticulata in Parkinson's disease. Mov. Disord. 15, 1064–1069 10.1002/1531-8257(200011)15:6%3C1064::AID-MDS1002%3E3.0.CO;2-C 11104187

[B52] Frechilla D. Cobreros A. Saldise L. Moratalla R. Insausti R. Luquin M. (2001). Serotonin 5-HT(1A) receptor expression is selectively enhanced in the striosomal compartment of chronic parkinsonian monkeys. Synapse 39, 288–296 10.1002/1098-2396(20010315)39:4<288::AID-SYN1011>3.0.CO;2-V 11169778

[B53] Gehlert D. R. Schober D. A. Gackenheimer S. L. Mais D. E. Ladouceur G. Robertson D. W. (1993). Synthesis and evaluation of [125I]-(S)-iodozacopride, a high affinity radioligand for 5HT3 receptors. Neurochem. Int. 23, 373–383 10.1016/0197-0186(93)90081-F 8220179

[B54] Gerard C. Martres M. P. Lefevre K. Miquel M. C. Verge D. Lanfumey L. (1997). Immuno-localization of serotonin 5-HT6 receptor-like material in the rat central nervous system. Brain Res. 746, 207–219 10.1016/S0006-8993(96)01224-3 9037500

[B55] Gerber R. Altar C. A. Liebman J. M. (1988). Rotational behavior induced by 8-hydroxy-DPAT, a putative 5HT-1A agonist, in 6-hydroxydopamine-lesioned rats. Psychopharmacology (Berl) 94, 178–182 10.1007/BF00176841 2965396

[B56] Goetz C. G. Damier P. Hicking C. Laska E. Müller T. Olanow C. W. (2007). Sarizotan as a treatment for dyskinesias in Parkinson's disease: double-blind placebo-controlled trial. Mov. Disord. 22, 179–186 10.1002/mds.21226 17094088

[B57] Goetz C. G. Laska E. Hicking C. Damier P. Muller T. Nutt J. (2008). Placebo influences on dyskinesia in Parkinson's disease. Mov. Disord. 23, 700–707 10.1002/mds.21897 18175337 PMC2689363

[B58] Guiard B. P. el Mansari M. Merali Z. Blier P. (2008). Functional interactions between dopamine, serotonin and norepinephrine neurons: an *in-vivo* electrophysiological study in rats with monoaminergic lesions. Int. J. Neuropsychopharmacol. 11, 625–639 10.1017/S1461145707008383 18205979

[B59] Guttman M. Boileau I. Warsh J. Saint-Cyr J. A. Ginovart N. Mccluskey T. (2007). Brain serotonin transporter binding in non-depressed patients with Parkinson's disease. Eur. J. Neurol. 14, 523–528 10.1111/j.1468-1331.2007.01727.x 17437611

[B60] Haapaniemi T. H. Ahonen A. Torniainen P. Sotaniemi K. A. Myllyla V. V. (2001). [123I]beta-CIT SPECT demonstrates decreased brain dopamine and serotonin transporter levels in untreated parkinsonian patients. Mov. Disord. 16, 124–130 10.1002/1531-8257(200101)16:1<124::AID-MDS1007>3.0.CO;2-R 11215571

[B61] Hall H. Farde L. Halldin C. Lundkvist C. Sedvall G. (2000). Autoradiographic localization of 5-HT(2A) receptors in the human brain using [(3)H]M100907 and [(11)C]M100907. Synapse 38, 421–431 10.1002/1098-2396(20001215)38:4<421::AID-SYN7>3.0.CO;2-X 11044889

[B62] Halliday G. M. Blumbergs P. C. Cotton R. G. Blessing W. W. Geffen L. B. (1990). Loss of brainstem serotonin- and substance P-containing neurons in Parkinson's disease. Brain Res. 510, 104–107 10.1016/0006-8993(90)90733-R 1691042

[B63] Hashimoto K. Kita H. (2008). Serotonin activates presynaptic and postsynaptic receptors in rat globus pallidus. J. Neurophysiol. 99, 1723–1732 10.1152/jn.01143.2007 18234984

[B64] Horisawa T. Ishiyama T. Ono M. Ishibashi T. Taiji M. (2013). Binding of lurasidone, a novel antipsychotic, to rat 5-HT7 receptor: analysis by [3H]SB-269970 autoradiography. Prog. Neuropsychopharmacol. Biol. Psychiatry 40, 132–137 10.1016/j.pnpbp.2012.08.005 23367506

[B65] Hoyer D. Pazos A. Probst A. Palacios J. M. (1986). Serotonin receptors in the human brain. II. Characterization and autoradiographic localization of 5-HT1C and 5-HT2 recognition sites. Brain Res. 376, 97–107 10.1016/0006-8993(86)90903-0 2941113

[B66] Huot P. Johnston T. H. Koprich J. B. Winkelmolen L. Fox S. H. Brotchie J. M. (2012a). Regulation of cortical and striatal 5-HT1A receptors in the MPTP-lesioned macaque. Neurobiol. Aging 33, 207.e9–e19 10.1016/j.neurobiolaging.2010.09.011 21051107

[B67] Huot P. Johnston T. H. Visanji N. P. Darr T. Pires D. Hazrati L. N. (2012b). Increased levels of 5-HT1A receptor binding in ventral visual pathways in Parkinson's disease. Mov. Disord. 27, 735–742 10.1002/mds.24964 22419526

[B68] Huot P. Johnston T. H. Winkelmolen L. Fox S. H. Brotchie J. M. (2012c). 5-HT2A receptor levels increase in MPTP-lesioned macaques treated chronically with L-DOPA. Neurobiol. Aging 33, 194.e5–e15 10.1016/j.neurobiolaging.2010.04.035 20561716

[B69] Iderberg H. Francardo V. Pioli E. Y. (2012). Animal models of L-DOPA-induced dyskinesia: an update on the current options. Neuroscience 211, 13–27 10.1016/j.neuroscience.2012.03.023 22465440

[B70] Inden M. Abe M. Minamino H. Takata K. Yoshimoto K. Tooyama I. (2012). Effect of selective serotonin reuptake inhibitors via 5-HT1A receptors on L-DOPA-induced rotational behavior in a hemiparkinsonian rat model. J. Pharmacol. Sci. 119, 10–19 10.1254/jphs.12003FP 22510520

[B71] Jakeman L. B. To Z. P. Eglen R. M. Wong E. H. Bonhaus D. W. (1994). Quantitative autoradiography of 5-HT4 receptors in brains of three species using two structurally distinct radioligands, [3H]GR113808 and [3H]BIMU-1. Neuropharmacology 33, 1027–1038 10.1016/0028-3908(94)90162-7 7845549

[B72] Jellinger K. (1990). New developments in the pathology of Parkinson's disease. Adv. Neurol. 53, 1–16 1978509

[B73] Karstaedt P. J. Kerasidis H. Pincus J. H. Meloni R. Graham J. Gale K. (1994). Unilateral destruction of dopamine pathways increases ipsilateral striatal serotonin turnover in rats. Exp. Neurol. 126, 25–30 10.1006/exnr.1994.1039 7512513

[B74] Kaya A. H. Vlamings R. Tan S. Lim L. W. Magill P. J. Steinbusch H. W. (2008). Increased electrical and metabolic activity in the dorsal raphe nucleus of Parkinsonian rats. Brain Res. 1221, 93–97 10.1016/j.brainres.2008.05.019 18565496

[B75] Kelland M. D. Freeman A. S. Chiodo L. A. (1990). Serotonergic afferent regulation of the basic physiology and pharmacological responsiveness of nigrostriatal dopamine neurons. J. Pharmacol. Exp. Ther. 253, 803–811 1971022

[B76] Kerenyi L. Ricaurte G. A. Schretlen D. J. McCann U. Varga J. Mathews W. B. (2003). Positron emission tomography of striatal serotonin transporters in Parkinson disease. Arch. Neurol. 60, 1223–1229 10.1001/archneur.60.9.1223 12975287

[B77] Kilpatrick G. J. Jones B. J. Tyers M. B. (1987). Identification and distribution of 5-HT3 receptors in rat brain using radioligand binding. Nature 330, 746–748 10.1038/330746a0 3696238

[B78] Kish S. J. Tong J. Hornykiewicz O. Rajput A. Chang L. J. Guttman M. (2008). Preferential loss of serotonin markers in caudate versus putamen in Parkinson's disease. Brain 131, 120–131 10.1093/brain/awm239 17956909

[B79] Kita H. Chiken S. Tachibana Y. Nambu A. (2007). Serotonin modulates pallidal neuronal activity in the awake monkey. J. Neurosci. 27, 75–83 10.1523/JNEUROSCI.4058-06.2007 17202474 PMC6672275

[B80] Kleedorfer B. Lees A. J. Stern G. M. (1991). Buspirone in the treatment of levodopa induced dyskinesias. J. Neurol. Neurosurg. Psychiatry 54, 376–377 10.1136/jnnp.54.4.376-a 2056334 PMC488507

[B81] Knobelman D. A. Kung H. F. Lucki I. (2000). Regulation of extracellular concentrations of 5-hydroxytryptamine (5-HT) in mouse striatum by 5-HT(1A) and 5-HT(1B) receptors. J. Pharmacol. Exp. Ther. 292, 1111–1117 10688630

[B82] Kohen R. Metcalf M. A. Khan N. Druck T. Huebner K. Lachowicz J. E. (1996). Cloning, characterization, and chromosomal localization of a human 5-HT6 serotonin receptor. J. Neurochem. 66, 47–56 10.1046/j.1471-4159.1996.66010047.x 8522988

[B83] Lagiere M. Navailles S. Mignon L. Roumegous A. Chesselet M. F. Deurwaerdere P. D. (2013). The enhanced oral response to the 5-HT_2_ agonist Ro 60-0175 in parkinsonian rats involves the entopeduncular nuclues: electrophysiological correlates. Exp. Brain Res. 230, 513–524 10.1007/s00221-013-3478-4 23535834

[B84] Lanfumey L. Hamon M. (2000). Central 5-HT(1A) receptors: regional distribution and functional characteristics. Nucl. Med. Biol. 27, 429–435 10.1016/S0969-8051(00)00107-4 10962246

[B85] Lavoie B. Parent A. (1990). Immunohistochemical study of the serotoninergic innervation of the basal ganglia in the squirrel monkey. J. Comp. Neurol. 299, 1–16 10.1002/cne.902990102 2212111

[B86] Liu J. Chu Y. X. Zhang Q. J. Wang S. Feng J. Li Q. (2007). 5,7-dihydroxytryptamine lesion of the dorsal raphe nucleus alters neuronal activity of the subthalamic nucleus in normal and 6-hydroxydopamine-lesioned rats. Brain Res. 1149, 216–222 10.1016/j.brainres.2007.02.052 17376410

[B87] Lopez-Gimenez J. F. Mengod G. Palacios J. M. Vilaro M. T. (2001). Regional distribution and cellular localization of 5-HT2C receptor mRNA in monkey brain: comparison with [3H]mesulergine binding sites and choline acetyltransferase mRNA. Synapse 42, 12–26 10.1002/syn.1095 11668587

[B88] Lucas G. Di Matteo V. De Deurwaerdere P. Porras G. Martin-Ruiz R. Artigas F. (2001). Neurochemical and electrophysiological evidence that 5-HT4 receptors exert a state-dependent facilitatory control *in vivo* on nigrostriatal, but not mesoaccumbal, dopaminergic function. Eur. J. Neurosci. 13, 889–898 10.1046/j.0953-816x.2000.01453.x 11264661

[B89] Maeda T. Kannari K. Shen H. Arai A. Tomiyama M. Matsunaga M. (2003). Rapid induction of serotonergic hyperinnervation in the adult rat striatum with extensive dopaminergic denervation. Neurosci. Lett. 343, 17–20 10.1016/S0304-3940(03)00295-7 12749987

[B90] Martin-Cora F. J. Pazos A. (2004). Autoradiographic distribution of 5-HT7 receptors in the human brain using [3H]mesulergine: comparison to other mammalian species. Br. J. Pharmacol. 141, 92–104 10.1038/sj.bjp.0705576 14656806 PMC1574165

[B91] Mengod G. Pompeiano M. Martinez-Mir M. I. Palacios J. M. (1990). Localization of the mRNA for the 5-HT2 receptor by *in situ* hybridization histochemistry. Correlation with the distribution of receptor sites. Brain Res. 524, 139–143 10.1016/0006-8993(90)90502-3 2400925

[B93] Mignon L. J. Wolf W. A. (2005). 8-hydroxy-2-(di-n-propylamino)tetralin reduces striatal glutamate in an animal model of Parkinson's disease. Neuroreport 16, 699–703 10.1097/00001756-200505120-00009 15858409

[B94] Miguelez C. Berrocoso E. Mico J. A. Ugedo L. (2013). l-DOPA modifies the antidepressant-like effects of reboxetine and fluoxetine in rats. Neuropharmacology 67, 349–358 10.1016/j.neuropharm.2012.11.016 23211937

[B95] Miguelez C. Grandoso L. Ugedo L. (2011). Locus coeruleus and dorsal raphe neuron activity and response to acute antidepressant administration in a rat model of Parkinson's disease. Int. J. Neuropsychopharmacol. 14, 187–200 10.1017/S146114571000043X 20426885

[B96] Minabe Y. Emori K. Ashby C. R. Jr. (1996). The depletion of brain serotonin levels by para-chlorophenylalanine administration significantly alters the activity of midbrain dopamine cells in rats: an extracellular single cell recording study. Synapse 22, 46–53 10.1002/(SICI)1098-2396(199601)22:1%3C46::AID-SYN5%3E3.3.CO;2-I 8822477

[B97] Mo J. Zhang H. Yu L. P. Sun P. H. Jin G. Z. Zhen X. (2010). L-stepholidine reduced L-DOPA-induced dyskinesia in 6-OHDA-lesioned rat model of Parkinson's disease. Neurobiol. Aging 31, 926–936 10.1016/j.neurobiolaging.2008.06.017 18707801

[B98] Nahimi A. Holtzermann M. Landau A. M. Simonsen M. Jakobsen S. Alstrup A. K. (2012). Serotonergic modulation of receptor occupancy in rats treated with L-DOPA after unilateral 6-OHDA lesioning. J. Neurochem. 120, 806–817 10.1111/j.1471-4159.2011.07598.x 22117574

[B99] Navailles S. Benazzouz A. Bioulac B. Gross C. De Deurwaerdere P. (2010a). High-frequency stimulation of the subthalamic nucleus and L-3,4-dihydroxyphenylalanine inhibit *in vivo* serotonin release in the prefrontal cortex and hippocampus in a rat model of Parkinson's disease. J. Neurosci. 30, 2356–2364 10.1523/JNEUROSCI.5031-09.2010 20147561 PMC6634027

[B100] Navailles S. Bioulac B. Gross C. De Deurwaerdere P. (2010b). Serotonergic neurons mediate ectopic release of dopamine induced by L-DOPA in a rat model of Parkinson's disease. Neurobiol. Dis. 38, 136–143 10.1016/j.nbd.2010.01.012 20096781

[B101] Navailles S. Lagiere M. Contini A. De Deurwaerdere P. (2013). Multisite intracerebral microdialysis to study the mechanism of L-DOPA induced dopamine and serotonin release in the parkinsonian brain. ACS Chem. Neurosci. 4, 680–692 10.1021/cn400046e 23541043 PMC3656765

[B102] Nevalainen N. Af Bjerken S. Lundblad M. Gerhardt G. A. Stromberg I. (2011). Dopamine release from serotonergic nerve fibers is reduced in L-DOPA-induced dyskinesia. J. Neurochem. 118, 12–23 10.1111/j.1471-4159.2011.07292.x 21534956 PMC3112269

[B103] Nirogi R. Kandikere V. Bhyrapuneni G. Saralaya R. Ajjala D. R. Aleti R. R. (2013). *In-vivo* rat striatal 5-HT4 receptor occupancy using non-radiolabelled SB207145. J. Pharm. Pharmacol. 65, 704–712 10.1111/jphp.12030 23600388

[B105] Oliver K. R. Kinsey A. M. Wainwright A. Sirinathsinghji D. J. (2000). Localization of 5-ht(5A) receptor-like immunoreactivity in the rat brain. Brain Res. 867, 131–142 10.1016/S0006-8993(00)02273-3 10837806

[B106] Olpe H. R. Koella W. P. (1977). The response of striatal cells upon stimulation of the dorsal and median raphe nuclei. Brain Res. 122, 357–360 10.1016/0006-8993(77)90302-X 837235

[B107] Palkovits M. Brownstein M. Saavedra J. M. (1974). Serotonin content of the brain stem nuclei in the rat. Brain Res. 80, 237–249 10.1016/0006-8993(74)90688-X 4424833

[B108] Park M. R. Gonzales-Vegas J. A. Kitai S. T. (1982). Serotonergic excitation from dorsal raphe stimulation recorded intracellularly from rat caudate-putamen. Brain Res. 243, 49–58 10.1016/0006-8993(82)91119-2 6214298

[B109] Pazos A. Cortes R. Palacios J. M. (1985). Quantitative autoradiographic mapping of serotonin receptors in the rat brain. II. Serotonin-2 receptors. Brain Res. 346, 231–249 10.1016/0006-8993(85)90857-1 4052777

[B110] Pazos A. Probst A. Palacios J. M. (1987). Serotonin receptors in the human brain–IV. Autoradiographic mapping of serotonin-2 receptors. Neuroscience 21, 123–139 10.1016/0306-4522(87)90327-7 3601071

[B111] Politis M. Wu K. Loane C. Kiferle L. Molloy S. Brooks D. J. (2010). Staging of serotonergic dysfunction in Parkinson's disease: an *in vivo* 11C-DASB PET study. Neurobiol. Dis. 40, 216–221 10.1016/j.nbd.2010.05.028 20594979

[B112] Pompeiano M. Palacios J. M. Mengod G. (1994). Distribution of the serotonin 5-HT2 receptor family mRNAs: comparison between 5-HT2A and 5-HT2C receptors. Brain Res. Mol. Brain Res. 23, 163–178 10.1016/0169-328X(94)90223-2 8028479

[B113] Prinz A. Selesnew L. M. Liss B. Roeper J. Carlsson T. (2013). Increased excitability in serotonin neurons in the dorsal raphe nucleus in the 6-OHDA mouse model of Parkinson's disease. Exp. Neurol. 248C, 236–245 10.1016/j.expneurol.2013.06.015 23810738

[B114] Prisco S. Esposito E. (1995). Differential effects of acute and chronic fluoxetine administration on the spontaneous activity of dopaminergic neurones in the ventral tegmental area. Br. J. Pharmacol. 116, 1923–1931 10.1111/j.1476-5381.1995.tb16684.x 8528581 PMC1909093

[B115] Querejeta E. Oviedo-Chavez A. Araujo-Alvarez J. M. Quinones-Cardenas A. R. Delgado A. (2005). *In vivo* effects of local activation and blockade of 5-HT1B receptors on globus pallidus neuronal spiking. Brain Res. 1043, 186–194 10.1016/j.brainres.2005.02.055 15862532

[B116] Radja F. Descarries L. Dewar K. M. Reader T. A. (1993). Serotonin 5-HT1 and 5-HT2 receptors in adult rat brain after neonatal destruction of nigrostriatal dopamine neurons: a quantitative autoradiographic study. Brain Res. 606, 273–285 10.1016/0006-8993(93)90995-Y 8490720

[B117] Raisman R. Cash R. Agid Y. (1986). Parkinson's disease: decreased density of 3H-imipramine and 3H-paroxetine binding sites in putamen. Neurology 36, 556–560 10.1212/WNL.36.4.556 2938025

[B118] Reijnders J. S. Ehrt U. Weber W. E. Aarsland D. Leentjens A. F. (2008). A systematic review of prevalence studies of depression in Parkinson's disease. Mov. Disord. 23, 183–189 10.1002/mds.21803 17987654

[B119] Reynolds G. P. Mason S. L. Meldrum A. De Keczer S. Parnes H. Eglen R. M. (1995). 5-Hydroxytryptamine (5-HT)4 receptors in post mortem human brain tissue: distribution, pharmacology and effects of neurodegenerative diseases. Br. J. Pharmacol. 114, 993–998 10.1111/j.1476-5381.1995.tb13303.x 7780656 PMC1510307

[B120] Riahi G. Morissette M. Levesque D. Rouillard C. Samadi P. Parent M. (2012). Effect of chronic l-DOPA treatment on 5-HT(1A) receptors in parkinsonian monkey brain. Neurochem. Int. 61, 1160–1171 10.1016/j.neuint.2012.08.009 22940695

[B121] Riahi G. Morissette M. Parent M. Di Paolo T. (2011). Brain 5-HT(2A) receptors in MPTP monkeys and levodopa-induced dyskinesias. Eur. J. Neurosci. 33, 1823–1831 10.1111/j.1460-9568.2011.07675.x 21501255

[B122] Riahi G. Morissette M. Samadi P. Parent M. Di Paolo T. (2013). Basal ganglia serotonin 1B receptors in parkinsonian monkeys with L-DOPA-induced dyskinesia. Biochem. Pharmacol. 86, 970–978 10.1016/j.bcp.2013.08.005 23954709

[B123] Rick C. E. Stanford I. M. Lacey M. G. (1995). Excitation of rat substantia nigra pars reticulata neurons by 5-hydroxytryptamine *in vitro*: evidence for a direct action mediated by 5-hydroxytryptamine2C receptors. Neuroscience 69, 903–913 10.1016/0306-4522(95)00283-O 8596658

[B124] Rozas G. Liste I. Guerra M. J. Labandeira-Garcia J. L. (1998). Sprouting of the serotonergic afferents into striatum after selective lesion of the dopaminergic system by MPTP in adult mice. Neurosci. Lett. 245, 151–154 10.1016/S0304-3940(98)00198-0 9605478

[B125] Rueter L. E. Tecott L. H. Blier P. (2000). *In vivo* electrophysiological examination of 5-HT2 responses in 5-HT2C receptor mutant mice. Naunyn Schmiedebergs Arch. Pharmacol. 361, 484–491 10.1007/s002109900181 10832601

[B126] Rylander D. Parent M. O'Sullivan S. S. Dovero S. Lees A. J. Bezard E. (2010). Maladaptive plasticity of serotonin axon terminals in levodopa-induced dyskinesia. Ann. Neurol. 68, 619–628 10.1002/ana.22097 20882603

[B127] Saavedra J. M. (1977). Distribution of serotonin and synthesizing enzymes in discrete areas of the brain. Fed. Proc. 36, 2134–2141 872947

[B128] Scatton B. Javoy-Agid F. Rouquier L. Dubois B. Agid Y. (1983). Reduction of cortical dopamine, noradrenaline, serotonin and their metabolites in Parkinson's disease. Brain Res. 275, 321–328 10.1016/0006-8993(83)90993-9 6626985

[B129] Shen K. Z. Johnson S. W. (2008). 5-HT inhibits synaptic transmission in rat subthalamic nucleus neurons *in vitro*. Neuroscience 151, 1029–1033 10.1016/j.neuroscience.2007.12.001 18248912 PMC2279230

[B130] Shen K. Z. Kozell L. B. Johnson S. W. (2007). Multiple conductances are modulated by 5-HT receptor subtypes in rat subthalamic nucleus neurons. Neuroscience 148, 996–1003 10.1016/j.neuroscience.2007.07.012 17706881 PMC2034448

[B131] Sinton C. M. Fallon S. L. (1988). Electrophysiological evidence for a functional differentiation between subtypes of the 5-HT1 receptor. Eur. J. Pharmacol. 157, 173–181 10.1016/0014-2999(88)90380-9 2906291

[B132] Stanford I. M. Kantaria M. A. Chahal H. S. Loucif K. C. Wilson C. L. (2005). 5-Hydroxytryptamine induced excitation and inhibition in the subthalamic nucleus: action at 5-HT(2C), 5-HT(4) and 5-HT(1A) receptors. Neuropharmacology 49, 1228–1234 10.1016/j.neuropharm.2005.09.003 16229866

[B133] Stanford I. M. Lacey M. G. (1996). Differential actions of serotonin, mediated by 5-HT1B and 5-HT2C receptors, on GABA-mediated synaptic input to rat substantia nigra pars reticulata neurons *in vitro*. J. Neurosci. 16, 7566–7573 8922413 10.1523/JNEUROSCI.16-23-07566.1996 PMC6579110

[B134] Stefani A. Surmeier D. J. Kitai S. T. (1990). Serotonin enhances excitability in neostriatal neurons by reducing voltage-dependent potassium currents. Brain Res. 529, 354–357 10.1016/0006-8993(90)90851-2 2282503

[B135] Steward L. J. Bufton K. E. Hopkins P. C. Davies W. E. Barnes N. M. (1993). Reduced levels of 5-HT3 receptor recognition sites in the putamen of patients with Huntington's disease. Eur. J. Pharmacol. 242, 137–143 10.1016/0014-2999(93)90073-Q 8253110

[B136] Takeuchi Y. Sawada T. Blunt S. Jenner P. Marsden C. D. (1991). Effects of 6-hydroxydopamine lesions of the nigrostriatal pathway on striatal serotonin innervation in adult rats. Brain Res. 562, 301–305 10.1016/0006-8993(91)90635-9 1685345

[B137] Tronci E. Lisci C. Stancampiano R. Fidalgo C. Collu M. Devoto P. (2013). 5-Hydroxy-tryptophan for the treatment of L-DOPA-induced dyskinesia in the rat Parkinson's disease model. Neurobiol. Dis. 60, 108–114 10.1016/j.nbd.2013.08.014 24004632

[B138] Ugedo L. Grenhoff J. Svensson T. H. (1989). Ritanserin, a 5-HT2 receptor antagonist, activates midbrain dopamine neurons by blocking serotonergic inhibition. Psychopharmacology (Berl) 98, 45–50 10.1007/BF00442004 2524859

[B139] Vandermaelen C. P. Bonduki A. C. Kitai S. T. (1979). Excitation of caudate-putamen neurons following stimulation of the dorsal raphe nucleus in the rat. Brain Res. 175, 356–361 10.1016/0006-8993(79)91016-3 487163

[B140] Vanover K. E. Betz A. J. Weber S. M. Bibbiani F. Kielaite A. Weiner D. M. (2008). A 5-HT2A receptor inverse agonist, ACP-103, reduces tremor in a rat model and levodopa-induced dyskinesias in a monkey model. Pharmacol. Biochem. Behav. 90, 540–544 10.1016/j.pbb.2008.04.010 18534670 PMC2806670

[B141] Varnas K. Halldin C. Hall H. (2004a). Autoradiographic distribution of serotonin transporters and receptor subtypes in human brain. Hum. Brain Mapp. 22, 246–260 10.1002/hbm.20035 15195291 PMC6872082

[B142] Varnas K. Thomas D. R. Tupala E. Tiihonen J. Hall H. (2004b). Distribution of 5-HT7 receptors in the human brain: a preliminary autoradiographic study using [3H]SB-269970. Neurosci. Lett. 367, 313–316 10.1016/j.neulet.2004.06.025 15337256

[B143] Varnas K. Halldin C. Pike V. W. Hall H. (2003). Distribution of 5-HT4 receptors in the postmortem human brain–an autoradiographic study using [125I]SB 207710. Eur. Neuropsychopharmacol. 13, 228–234 10.1016/S0924-977X(03)00009-9 12888181

[B144] Wang H. Chen X. Y. Chen W. F. Xue Y. Wei L. Chen L. (2013). Anticataleptic effects of 5-HT1B receptors in the globus pallidus. Neurosci. Res. 77, 162–169 10.1016/j.neures.2013.09.002 24045116

[B145] Wang S. Zhang Q. J. Liu J. Wu Z. H. Wang T. Gui Z. H. (2009). Unilateral lesion of the nigrostriatal pathway induces an increase of neuronal firing of the midbrain raphe nuclei 5-HT neurons and a decrease of their response to 5-HT(1A) receptor stimulation in the rat. Neuroscience 159, 850–861 10.1016/j.neuroscience.2008.12.051 19174182

[B146] Wilms K. Vierig G. Davidowa H. (2001). Interactive effects of cholecystokinin-8S and various serotonin receptor agonists on the firing activity of neostriatal neuronesin rats. Neuropeptides 35, 257–270 10.1054/npep.2001.0875 12030810

[B147] Wong E. H. Reynolds G. P. Bonhaus D. W. Hsu S. Eglen R. M. (1996). Characterization of [3H]GR 113808 binding to 5-HT4 receptors in brain tissues from patients with neurodegenerative disorders. Behav. Brain Res. 73, 249–252 10.1016/0166-4328(96)00106-4 8788512

[B148] Xiang Z. Wang L. Kitai S. T. (2005). Modulation of spontaneous firing in rat subthalamic neurons by 5-HT receptor subtypes. J. Neurophysiol. 93, 1145–1157 10.1152/jn.00561.2004 15738272

[B149] Yakel J. L. Trussell L. O. Jackson M. B. (1988). Three serotonin responses in cultured mouse hippocampal and striatal neurons. J. Neurosci. 8, 1273–1285 2965756 10.1523/JNEUROSCI.08-04-01273.1988 PMC6569259

[B150] Yamada H. Aimi Y. Nagatsu I. Taki K. Kudo M. Arai R. (2007). Immunohistochemical detection of L-DOPA-derived dopamine within serotonergic fibers in the striatum and the substantia nigra pars reticulata in Parkinsonian model rats. Neurosci. Res. 59, 1–7 10.1016/j.neures.2007.05.002 17586078

[B151] Zhang Q. J. Gao R. Liu Y. P. Wang S. (2007a). Changes in the firing activity of serotonergic neurons in the dorsal raphe nucleus in a rat model of Parkinson's disease. Sheng Li Xue Bao 59, 183–189 17437041

[B152] Zhang X. Andren P. E. Svenningsson P. (2007b). Changes on 5-HT(2) receptor mRNAs in striatum and subthalamic nucleus in Parkinson's disease model. Physiol. Behav. 92, 29–33 10.1016/j.physbeh.2007.05.033 17588622

[B153] Zhang S. J. Wang H. Xue Y. Yung W. H. Chen L. (2010). Behavioral and electrophysiological effects of 5-HT in globus pallidus of 6-hydroxydopamine lesioned rats. J. Neurosci. Res. 88, 1549–1556 10.1002/jnr.22325 20029979

[B154] Zhang X. Andren P. E. Greengard P. Svenningsson P. (2008). Evidence for a role of the 5-HT1B receptor and its adaptor protein, p11, in L-DOPA treatment of an animal model of Parkinsonism. Proc. Natl. Acad. Sci. U.S.A. 105, 2163–2168 10.1073/pnas.0711839105 18256188 PMC2538893

[B155] Zhou F. C. Bledsoe S. Murphy J. (1991). Serotonergic sprouting is induced by dopamine-lesion in substantia nigra of adult rat brain. Brain Res. 556, 108–116 10.1016/0006-8993(91)90553-8 1718555

